# Spinal muscular atrophy with five *SMN2* copies: Phenotypic heterogeneity and implications for newborn screening and treatment decisions

**DOI:** 10.3389/fneur.2026.1898311

**Published:** 2026-07-30

**Authors:** Olesja Parmova, Blanka Adamova

**Affiliations:** 1 Department of Neurology, Centre for Neuromuscular Diseases (Associated National Centre in the ERN EURO-NMD), University Hospital Brno, Brno, Czechia; 2 Faculty of Medicine, Masaryk University, Brno, Czechia

**Keywords:** genotype–phenotype correlation, newborn screening, phenotypic heterogeneity, presymptomatic SMA, *SMN2* copy number, spinal muscular atrophy

## Abstract

**Background:**

The severity of spinal muscular atrophy (SMA) is partially influenced by *SMN2* copy number, although this relationship becomes less reliable at higher copy numbers. The implementation of newborn screening has led to increasing identification of presymptomatic infants with five *SMN2* copies, a subgroup for which long-term clinical outcomes remain poorly defined. Consequently, treatment decisions regarding immediate intervention versus careful clinical observation remain challenging.

**Methods:**

We describe three patients with genetically confirmed SMA (each with homozygous *SMN1* deletion and five *SMN2* copies).

**Results:**

Marked phenotypic variability was observed. Two siblings identified through family screening showed divergent clinical courses despite a shared genetic background, with one developing adult-onset proximal weakness; the other remains asymptomatic. A third patient had adolescent-onset disease progressing to severe motor impairment. These findings highlight the limited prognostic value of *SMN2* copy number.

**Conclusion:**

Patients with five *SMN2* copies represent a clinically heterogeneous group requiring careful longitudinal monitoring. Our findings highlight the limitations of *SMN2* copy number as an independent prognostic marker and illustrate the growing challenges of treatment decision-making and therapy timing in the era of newborn screening.

## 1 Introduction

Spinal muscular atrophy (SMA) is an autosomal recessive neuromuscular disorder caused by homozygous deletions or mutations in the *SMN1* gene, leading to reduced levels of survival motor neuron (SMN) protein. The number of copies of the paralogous *SMN2* gene is a major disease modifier and is widely used as a prognostic marker, with lower copy numbers generally associated with more severe phenotypes (1).

However, this genotype–phenotype correlation becomes less reliable at higher *SMN2* copy numbers. Patients with four or more *SMN2* copies show substantial clinical variability, and the predictive value of *SMN2* copy number in this subgroup is limited (2). This variability is probably influenced by additional genetic and non-genetic modifiers beyond *SMN2* copy number (3, 4). In particular, individuals with five *SMN2* copies remain poorly characterised, with only scarce data available regarding long-term natural history, phenotypic spectrum, and clinical outcomes.

The introduction of newborn screening and disease-modifying therapies has introduced new challenges to clinical management of these patients. While early treatment is clearly beneficial in patients with lower *SMN2* copy numbers (5), the optimal management of asymptomatic individuals with five *SMN2* copies remains uncertain. Immediate treatment is generally recommended for infants with two to four *SMN2* copies, whereas management of those with five copies remains controversial. This uncertainty has become particularly relevant with the implementation of newborn screening programmes, where clinicians are now confronted with presymptomatic infants carrying five *SMN2* copies and the absence of clear evidence-based guidance regarding immediate treatment initiation versus careful observation. This lack of long-term observational data in this subgroup further complicates clinical decision-making, as treatment decisions may need to be made before symptom onset. In this context, it is often difficult to distinguish between the natural course of a typically mild, late-onset phenotype and the potential effect of early therapeutic intervention.

In this study, we present a case series of three patients, each with genetically confirmed SMA and five *SMN2* copies, illustrating marked phenotypic variability, including intrafamilial differences, and discuss the implications for clinical decision-making.

## 2 Methods and results

Three patients, each with genetically confirmed SMA (homozygous *SMN1* deletion, five *SMN2* copies), were included. *SMN1* deletion and *SMN2* copy number were determined using multiplex ligation-dependent probe amplification (MLPA) in the accredited molecular genetics laboratory of a university hospital with extensive expertise in SMA diagnostics, which is part of a European Reference Network (ERN) neuromuscular centre. Clinical characteristics are summarized in Table 1. Two patients were siblings identified through family screening; the third patient was diagnosed based on clinical presentation. Despite identical *SMN2* copy numbers, marked phenotypic variability was observed. All patients were followed at the same neuromuscular centre and informed consent was obtained from each patient.

**Table 1:** Clinical characteristics of patients with five *SMN2* copies.

Patient	Sex	Current age	Age at onset	Mode of detection	Ambulation	Clinical phenotype	EMG findings	Treatment
1	M	42	30 years	Family screening	Fully ambulant	Mild adult-onset proximal weakness	Chronic neurogenic changes with ongoing denervation	Nusinersen
2	F	44	Asymptomatic	Family screening	Fully ambulant	No clinical weakness	Mild chronic neurogenic changes, no denervation	None (follow-up)
3	M	68	14 years	Clinical presentation	Ambulant with bilateral support	Adolescent-onset severe proximal weakness	Chronic neurogenic changes with infrequent denervation	None (limited treatment adherence)

Patient 1 developed slowly progressive proximal lower limb weakness in adulthood (around 30 years of age), initially manifesting as difficulty rising from a squat. Additional symptoms included difficulty performing hip flexion while standing, such as when putting on shoes and occasional episodes of knee instability during walking. He was diagnosed through family screening following identification of SMA in a relative, rather than on clinical presentation. Neurological examination revealed normal strength in the upper limbs (Medical Research Council (MRC) grade 5/5 in all muscle groups) and asymmetric proximal weakness in the lower limbs, more pronounced on the right side (hip flexion MRC 3− right, 3 left; knee extension MRC 4 bilaterally), with normal distal strength. Functional assessment demonstrated normal performance, with Revised Upper Limb Module (RULM) score of 37, Hammersmith Functional Motor Scale Expanded (HFMSE) score of 66, and 6-Minute Walk test (6MWT) distance of 650 m. Pulmonary function was within normal limits [forced vital capacity (FVC) 108% predicted, maximal inspiratory pressure (MIP) 90% predicted, and maximal expiratory pressure (MEP) 88% predicted]. Nerve conduction studies were normal. Needle EMG showed marked chronic neurogenic changes with ongoing denervation and reinnervation. Denervation was present in the tibialis anterior muscle; pronounced chronic neurogenic changes were observed in both proximal (vastus lateralis muscle and vastus medialis muscle) and distal muscles (tibialis anterior). Serum creatine kinase was mildly elevated (11 μkat/L), and myoglobin was almost normal (82 μg/L). Muscle MRI revealed subtle fatty infiltration consistent with early muscle involvement, predominantly affecting the quadriceps muscles bilaterally (more pronounced on the right) and the right tibialis anterior muscle. Nusinersen therapy was initiated because the patient had clinically evident proximal muscle weakness, neurogenic EMG abnormalities including active denervation, and functional impairment consistent with symptomatic SMA. The EMG findings supported the presence of ongoing disease activity rather than a purely static deficit, favouring initiation of disease-modifying treatment.

Patient 2, the sibling of Patient 1, was also identified through family screening. She reported no functional limitations in daily activities or sports. Neurological examination revealed normal muscle strength across all muscle groups (MRC 5/5), and she was able to perform repeated squats without difficulty. Serum muscle enzymes were within normal limits. Functional SMA-specific scales were not performed, as no clinical deficits were evident. Needle EMG, however, revealed mild chronic neurogenic changes without ongoing denervation. These abnormalities were limited to proximal muscles (vastus lateralis and vastus medialis), while distal muscles showed no neurogenic changes. As the patient remained clinically asymptomatic with normal functional examination despite subtle neurogenic EMG findings, a strategy of careful clinical follow-up without immediate treatment was adopted after multidisciplinary discussion and shared decision-making with the patient.

Patient 3 was referred for evaluation based on clinical and electrophysiological suspicion of a neuromuscular disorder. The patient reported symptom onset at approximately 14 years of age, initially presenting with reduced physical performance during school activities and difficulty rising from a squat. During late adolescence, he experienced increasing upper limb weakness, including progressive loss of the ability to perform previously achievable physical and sports activities. He reported significant functional limitations during compulsory military service, which he was able to complete only with considerable difficulty. He remained ambulant without support until approximately 50 years of age, followed by gradual progression to severe motor impairment. At last evaluation, neurological examination revealed pronounced muscle atrophy affecting both upper and lower limb girdle muscles. In the upper limbs, there was severe proximal weakness (shoulder abduction MRC 3− right, 2 left; elbow flexion 3+ right, 3− left; elbow extension 4− right, 3+ left), with relatively preserved distal strength (MRC 4 bilaterally). In the lower limbs, there was severe proximal weakness (hip flexion MRC 1, knee extension 2), with only mild distal involvement (ankle dorsiflexion and plantar flexion MRC 4). Deep tendon reflexes were absent in the upper limbs and reduced or absent in the lower limbs. Pulmonary function testing showed forced vital capacity within the lower range of normal (FVC 79% predicted in the sitting position), without significant decline in the supine position. Due to the onset of symptoms in adolescence and the very slow disease progression, a hereditary myopathy, such as limb-girdle muscular dystrophy, was initially considered. However, prior EMG findings, including chronic neurogenic changes in both proximal and distal muscles of the upper and lower limbs and infrequent denervation changes, suggested a neurogenic process, which led to the decision to perform genetic testing for SMA. Genetic confirmation of SMA with five *SMN2* copies was an unexpected finding. No disease-modifying therapy was initiated due to significant comorbidities and limited treatment adherence.

## 3 Discussion

Our case series highlights the marked phenotypic heterogeneity associated with five *SMN2* copies, a subgroup for which long-term untreated natural history remains poorly characterised. Although patients with five *SMN2* copies have been reported within larger SMA cohorts, detailed longitudinal clinical descriptions of their phenotypic spectrum remain limited. Adult patients with five *SMN2* copies may provide important insight into the broader phenotypic spectrum and long-term disease course of this currently understudied subgroup. Although a higher *SMN2* copy number is generally associated with a milder phenotype, this correlation is not deterministic, particularly at higher copy numbers and at the upper end of the clinical spectrum (2). In our cohort, identical *SMN2* copy numbers were associated with markedly different phenotypes, ranging from a clinically asymptomatic individual with only subtle needle EMG abnormalities, through mild adult-onset weakness, to severe long-term phenotype originating in adolescence. The discordant presentation in two siblings further supports the role of additional modifying factors beyond *SMN2* copy number, including genetic, epigenetic, and possibly environmental influences (3, 4). These findings are consistent with previous work showing that genotype–phenotype correlations become less reliable as *SMN2* copy number increases (2). Further studies are needed to identify reliable prognostic biomarkers that could improve prediction of disease course beyond *SMN2* copy number alone.

An important observation is that a clinically mild disease may remain unrecognised for many years despite clear evidence of motor neuron involvement. Such a pattern is consistent with the natural history of adult-onset SMA, where progression may be slow and diagnosis delayed (6). In addition, slowly progressive proximal weakness with long disease duration may initially suggest hereditary myopathies, highlighting the importance of electrophysiological evaluation in guiding diagnosis.

Our findings suggest that standard functional scales may have limited sensitivity in patients at the mild end of the SMA spectrum. In Patient 1, clinically relevant weakness and functional complaints were present despite ceiling scores on HFMSE, RULM, and 6MWT. This ceiling effect is well recognised and has been described in adult SMA cohorts, particularly in ambulant and high-functioning patients, and limits the ability of these tools to detect subtle impairment or early progression (7). In our case, this discrepancy was further supported by both electromyography and muscle MRI, which demonstrated neurogenic changes and early fatty infiltration despite preserved functional performance. Our findings therefore support the need for complementary tools in mildly affected individuals.

In contrast, electrophysiological assessment appeared sensitive across the phenotypic spectrum. Patient 1 showed clear chronic neurogenic changes with ongoing denervation despite mild clinical involvement. Patient 2 demonstrated chronic neurogenic abnormalities despite being clinically asymptomatic. These findings suggest that EMG may detect subclinical disease in individuals with five *SMN2* copies and may represent a valuable adjunct in both diagnosis and follow-up, particularly when clinical examination and functional scales remain inconclusive.

These observations are especially relevant in the era of newborn screening and disease-modifying therapies, where increasing numbers of presymptomatic individuals with five *SMN2* copies are being identified before the onset of clinical symptoms. Current management of individuals with five and more *SMN2* copies remains one of the least well-defined areas in SMA care, as reliable prognostic markers allowing accurate prediction of disease severity and optimal timing of treatment initiation remain lacking. Management approaches vary across countries and centres (8). Current recommendations differ internationally, with some expert groups favouring careful observation and regular follow-up for infants with five *SMN2* copies, whereas others advocate a lower threshold for treatment initiation because of concerns regarding irreversible motor neuron loss before symptom recognition. Recent consensus statements also emphasise the complexity of interpreting *SMN2* copy number in clinical practice (9). Although early treatment is beneficial in patients with fewer *SMN2* copies, its role in individuals with higher copy numbers remains uncertain. Available presymptomatic data in patients with four or more *SMN2* copies remain limited by relatively short follow-up, making it difficult to distinguish treatment effects from natural disease course (10). Therefore, interpretation of these data remains difficult. As a result, clinicians are frequently required to balance the potential benefits of very early treatment against the possibility of prolonged asymptomatic or minimally symptomatic disease.

Our findings also illustrate why counselling families after newborn screening can be particularly challenging in this subgroup. The three patients described here shared the same *SMN2* copy number but exhibited markedly different outcomes, ranging from an asymptomatic adult with only subtle electrophysiological abnormalities to severe lifelong motor disability. If identified through newborn screening, all three individuals would have been classified as presymptomatic infants with five *SMN2* copies and would have appeared genetically indistinguishable at birth. However, their subsequent clinical trajectories differed substantially. These observations highlight the limitations of relying on *SMN2* copy number alone when estimating prognosis and emphasise the need for additional biomarkers capable of predicting disease severity and treatment responsiveness.

These findings support an individualised approach based on longitudinal clinical assessment rather than *SMN2* copy number alone. They also highlight the need for prognostic biomarkers beyond *SMN2* copy number to support treatment decisions in presymptomatic individuals. They further suggest that long-term observational data from untreated or minimally affected individuals remain essential for refining treatment algorithms in the newborn screening era.

This study has limitations, including its retrospective design, small sample size, and incomplete longitudinal data in some patients. Another limitation is that known genetic modifiers of disease severity were not systematically investigated in our patients. Therefore, the contribution of these modifiers to the observed phenotypic heterogeneity remains unknown. In addition, the depth of available clinical data differed between patients because of the retrospective nature of the study. In particular, comprehensive muscle MRI, longitudinal respiratory assessments, and serial laboratory measurements were not available for all individuals, especially Patient 3, limiting detailed phenotypic comparisons. However, the study provides clear evidence of phenotypic variability, including intrafamilial differences, within a genetically homogeneous subgroup.

## 4 Conclusion

In conclusion, individuals with five *SMN2* copies poses a clinically heterogeneous subgroup of SMA with highly variable age at onset and disease severity. In the era of newborn screening, this heterogeneity represents substantial challenges for prognostic counselling and treatment decisions. Our observations demonstrate that *SMN2* copy number alone is insufficient to predict long-term clinical outcome and support the need for careful longitudinal monitoring and the development of additional prognostic biomarkers to guide management of presymptomatic individuals identified through newborn screening.

## Data Availability

The original contributions presented in the study are included in the article. Further inquiries can be directed to the corresponding author.
